# 808 Resource Support to Optimize Recovery (RESTORE) Document Study: Aftercare Resources for Burn Survivors

**DOI:** 10.1093/jbcr/iraf019.339

**Published:** 2025-04-01

**Authors:** Renee Noordzij, Camille Carnevale, Diana Tenney-Laperriere, Carla Tierney-Hendricks, Lewis Kazis, Colleen Ryan, Amy Acton, Mary Slavin, Jeffrey Schneider

**Affiliations:** Spaulding Rehabilitation Hospital; Massachusetts General Hospital Institute of Health Professions; BH-BIMS; Spaulding Rehabilitation Hospital; Boston University; Shriners Children’s - Boston and Massachusetts General Hospital; Phoenix Society for Burn Survivors; Spaulding Rehabilitation Hospital; Spaulding Rehabilitation Hospital, Harvard Medical School

## Abstract

**Introduction:**

Adverse sequelae due to burn injury may be addressed by resources provided by healthcare institutions, however, burn survivors commonly report a lack of resources and assistance after hospitalization. Therefore, the aim of this study is to identify and categorize the resources provided by healthcare institutions to assist burn survivors in their post-discharge transition to the aftercare phase of recovery.

**Methods:**

Burn injury healthcare providers were recruited nationally to participate in this document study. Selected representatives provided data on their institutions’ burn aftercare resources and implementation strategies. Inclusion criteria for resources were: related to adult burn survivors, related to post-acute care or community reintegration, and introduced in inpatient or outpatient settings. The International Classification of Functioning, Disability, and Health (ICF) Core Set for burn injury provided the conceptual framework to deductively code and analyze the resources provided. The resource provider as well as when, where, and how the resources are provided were documented by the selected representatives. The implementation of resources was then inductively coded and analyzed.

**Results:**

A total of 14 healthcare institutions provided data on 255 resources. Resources addressed various ICF domains as follows: 49% were related to Environmental Factors, 39.2% were related to Activities and Participation, and 11.8% were related to Body Functions. The primary topics covered included health services, professionals, social security services, emotional recovery, self-management, and wound care. Important missing topics were fatigue, sleep, respiratory functioning, and thermoregulation. Analysis of resource implementation demonstrated a reliance on social workers and nurses. Resource formats commonly used were handouts, packets, or verbal communication. Most resources were provided to inpatients, primarily during the discharge process.

**Conclusions:**

This document study provides an overview of the burn aftercare resources currently provided by healthcare institutions in the U.S. Strengths and gaps in burn aftercare resource content were noted, as well as trends in the implementation of these resources.

**Applicability of Research to Practice:**

Burn centers can utilize these findings to enhance the comprehensiveness of the current aftercare resources they provide. This study will inform future guidelines and implementation strategies for resource distribution to burn survivors.

**Funding for the Study:**

NIDILRR #90DPBU0008. Partial Support was obtained from Shriners Hospitals for Children (Grant #79136-BOS-23 and 79138-BOS-23).

